# Heme Oxygenase-1 May Affect Cell Signalling via Modulation of Ganglioside Composition

**DOI:** 10.1155/2018/3845027

**Published:** 2018-09-19

**Authors:** Václav Šmíd, Jakub Šuk, Neli Kachamakova-Trojanowska, Jana Jašprová, Petra Valášková, Alicja Józkowicz, Józef Dulak, František Šmíd, Libor Vítek, Lucie Muchová

**Affiliations:** ^1^Institute of Medical Biochemistry and Laboratory Diagnostics, 1st Faculty of Medicine and General University Hospital in Prague, Charles University, Katerinska 32, 12108 Prague, Czech Republic; ^2^4th Department of Internal Medicine, 1st Faculty of Medicine and General University Hospital in Prague, Charles University, U Nemocnice 499/2, 12801 Prague, Czech Republic; ^3^Department of Medical Biotechnology, Faculty of Biochemistry, Biophysics and Biotechnology, Jagiellonian University, 7 Gronostajowa St., 30-387 Krakow, Poland; ^4^Malopolska Centre for Biotechnology, Jagiellonian University, Gronostajowa str 7a, 30-387 Krakow, Poland

## Abstract

Heme oxygenase 1 (Hmox1), a ubiquitous enzyme degrading heme to carbon monoxide, iron, and biliverdin, is one of the cytoprotective enzymes induced in response to a variety of stimuli, including cellular oxidative stress. Gangliosides, sialic acid-containing glycosphingolipids expressed in all cells, are involved in cell recognition, signalling, and membrane stabilization. Their expression is often altered under many pathological and physiological conditions including cell death, proliferation, and differentiation. The aim of this study was to assess the possible role of Hmox1 in ganglioside metabolism in relation to oxidative stress. The content of liver and brain gangliosides, their cellular distribution, and mRNA as well as protein expression of key glycosyltransferases were determined in *Hmox1* knockout mice as well as their wild-type littermates. To elucidate the possible underlying mechanisms between Hmox1 and ganglioside metabolism, hepatoblastoma HepG2 and neuroblastoma SH-SY5Y cell lines were used for *in vitro* experiments. Mice lacking *Hmox1* exhibited a significant increase in concentrations of liver and brain gangliosides and in mRNA expression of the key enzymes of ganglioside metabolism. A marked shift of GM1 ganglioside from the subsinusoidal part of the intracellular compartment into sinusoidal membranes of hepatocytes was shown in *Hmox1* knockout mice. Induction of oxidative stress by chenodeoxycholic acid *in vitro* resulted in a significant increase in GM3, GM2, and GD1a gangliosides in SH-SY5Y cells and GM3 and GM2 in the HepG2 cell line. These changes were abolished with administration of bilirubin, a potent antioxidant agent. These observations were closely related to oxidative stress-mediated changes in sialyltransferase expression regulated at least partially through the protein kinase C pathway. We conclude that oxidative stress is an important factor modulating synthesis and distribution of gangliosides *in vivo* and *in vitro* which might affect ganglioside signalling in higher organisms.

## 1. Introduction

Heme oxygenase 1 (Hmox1) is a highly inducible antioxidant and cytoprotective enzyme in the heme catabolic pathway generating equimolar amounts of iron, carbon monoxide, and biliverdin which is immediately reduced to bilirubin [1]. Hmox1 activity—also due to the effect of its bioactive products—affects pathophysiology of many neurologic, cardiovascular, and pulmonary diseases [2–4]. In the liver, Hmox1 plays an important role in hepatic fat accumulation, fibrogenesis, ischemia-reperfusion, and oxidative injury [5]. Moreover, upon *Hmox1* knockout, the cells and/or animals become more vulnerable to oxidative stress. Free radical formation as well as oxidative stress-associated cytotoxicity are increased in *Hmox1* knockouts due to reduced antioxidant bilirubin and vasoactive carbon monoxide formation, disruption of iron homeostasis, and accumulation of prooxidative heme [6]. Due to iron accumulation, liver is one of the tissues most affected by an increased oxidative stress in *Hmox1* knockout mice and increased lipid peroxidation, fibrosis, and hepatic injury have been described in these animals [5]. Furthermore, an increase in some key cytoprotective genes such as NAD(P)H dehydrogenase quinone 1 and glutathione S-transferase P1 and marked decrease in peroxyl radical scavenging activity have been described in *Hmox1* knockouts even under basal (unstimulated) conditions [7]. Bilirubin per se is considered a potent endogenous antioxidant protecting against diseases associated with oxidative stress [8] and counteracting harmful effects of various prooxidants including hydrophobic bile acids (BA) on cells and tissues [9]. In fact, both bilirubin and BA are accumulated in plasma and tissues during cholestasis and while BA are responsible for increased lipid peroxidation and oxidative liver damage, bilirubin has a protective effect [10].

Gangliosides are ubiquitously found in all tissues, but most abundantly in the nervous system [11]. They substantially influence the organization of the membrane and the function of specific membrane-associated proteins due to lipid-lipid and lipid-protein lateral interactions [12]. In the brain, ganglioside expression correlates with neurogenesis, synaptogenesis, synaptic transmission, and cell proliferation [13, 14].

It is known that gangliosides form so called caveolae or “detergent resistant microdomains” (DRM), which are crucial elements for cell-cell recognition, adhesion, and especially membrane stabilization [15, 16]. There is also evidence that caveolin-1, an important component of caveolae, interacts with Hmox1, modulates its activity, and can act as a natural competitive inhibitor of Hmox1 with heme [17]. Moreover, gangliosides have been found to inhibit hydroxyl radical formation *in vitro* [18] and also modulate ROS formation in human leukocytes [19] and neuronal cells [20].

Despite the close relationship of gangliosides and Hmox1 in DRM, there are only few reports discussing the possible role of Hmox1 or oxidative stress in ganglioside metabolism [21, 22]. The aim of this study was to assess the role of *Hmox1* knockout and associated oxidative stress on ganglioside metabolism and to identify the possible underlying mechanisms.

## 2. Materials and Methods

### 2.1. Materials

Paraformaldehyde, biotin, bovine serum albumin (BSA), phorbol 12-myristate 13-acetate (protein kinase C (PKC) activator), Ro 31-0432 (PKC inhibitor), chenodeoxycholic acid (CDCA), diaminobenzidine tetrahydrochloride tablets, NADPH, and sulfosalicylic acid were supplied by Sigma-Aldrich (St. Louis, MO, USA); avidin was obtained from Fluka (Buchs, Switzerland), the cholera toxin B subunit (CTB) peroxidase conjugated came from List Biological Laboratories (CA, USA), and the HPTLC silica-gel plates came from Merck (Darmstadt, Germany). Cell plates were supplied by Corning (NY, USA). The TaqMan® Gene Expression Master Mix, High-Capacity RNA-to-cDNA Kit, and the TaqMan Gene Expression Assay kit for mouse and human genes were obtained from Life Technologies (Carlsbad, CA, USA). The QIAshredder kit and RNeasy Plus Mini Kit were supplied by Qiagen (USA). All other chemicals were purchased locally from Penta (Prague, Czech Republic).

### 2.2. Animals

*Hmox1* ^−/−^ (*n* = 9; KO—knockout) mice and *Hmox1*^+/+^ (*n* = 6; Wt—wild type) littermates (C57Bl/6xFVB, 8-week-old males) were used for all the experiments. Breeding heterozygote pairs of *Hmox1*-deficient mice were initially kindly provided by Anupam Agarwal, University of Alabama (Birmingham, AL). The *Hmox1^−/−^* strain poorly breeds on pure C57/Bl6 background (5.1% of expected *Hmox1^−/−^* pups) and therefore is maintained on mixed C57/Bl6 × FVB background (20.1% of expected *Hmox^−/−^* pups, when *Hmox^−/−^* males are crossed with *Hmox^+/−^* females) [23]. All *Hmox1^+/+^* controls were C57/Bl6xFVB littermates from the same breeders used to obtain *Hmox1^−/−^* mice. They had free access to food and water and were kept in individually ventilated cages with a 12:12 day/night cycle, under a specific pathogen-free regime. All aspects of the study met the accepted criteria of experimental use of laboratory animals, and all protocols were approved by the Animal Research Committee of the 1st Faculty of Medicine, Charles University, Prague, Czech Republic, and by the 1st Local Ethics Committee for Animal Research, Krakow, Poland.

### 2.3. Tissue Preparation

Mice were intraperitoneally anesthetized with ketamine (90 mg/kg) and xylazine (10 mg/kg) and sacrificed by cervical dislocation at day 5. The inferior vena cava was cannulated through laparotomy, and blood samples were collected, transferred to EDTA-containing tubes, mixed, and placed on ice. An aliquot was centrifuged to separate out the plasma. The livers and brains were then harvested and weighed. Pieces of liver tissues were appropriately processed for further biochemical and histochemical analyses (see below). For quantitative histochemical analysis of GM1 ganglioside, the liver specimens were collected using a systematic uniform random sampling method [24].

For the RNA analysis, 100 mg of tissue was immediately placed in 1.5 mL microcentrifuge tubes containing RNAlater (Qiagen, Valencia, CA, USA). The tubes were stored at −80°C until total RNA isolation.

### 2.4. Extraction and TLC Densitometry of Liver and Brain Gangliosides

The chloroform-methanol extraction of gangliosides from the liver tissue was used—the procedure previously described by Majer et al. [25]—and gangliosides were finally purified on a small silica gel column [26]. Brain gangliosides were isolated as described previously [27, 28]. All ganglioside samples were separated in a solvent system (chloroform/methanol/0.2% aqueous CaCl_2_, 55/45/10, *v*/*v*/*v*) and detected with resorcinol-HCl reagent. The densitometry was performed according to Majer et al. [25] using a CATS3 Software, CAMAG (Switzerland).

GSL are abbreviated according to recommendations of the IUPAC-IUB Commission on Biochemical Nomenclature [29]: glycosyltransferases: *GlcT*, UDP-glucose ceramide glucosyltransferase; *GalTI*, UDP-Gal:betaGlcNAc beta-1,4-galactosyltransferase; *ST3GalV (GM3 synthase)*, ST3 beta-galactoside alpha-2,3-sialyltransferase 5; *ST8SiaI (GD3 synthase)*, ST8 alpha-*N*-acetylneuraminide alpha-2,8-sialyltransferase 1; *B4GalNTI (GM2/GD2 synthase)*, beta-1,4-*N*-acetyl-galactosaminyltransferase 1; and *B3GalTIV (GM1 synthase)*, UDP-Gal:betaGlcNAc beta 1,3-galactosyltransferase.

### 2.5. GM1 Histochemistry

GM1 was determined using a modified procedure according to Jirkovská et al. [30]. In brief, 4% formaldehyde was freshly prepared by depolymerization of paraformaldehyde (pH = 7.2). Frozen 6 *μ*m thin sections were first fixed in dry cold acetone (−20°C) for 15 min and then in 4% freshly prepared paraformaldehyde for 5 min. Endogenous peroxidase activity was blocked by incubation for 15 min in phosphate-buffered saline (PBS) supplemented by 1% H_2_O_2_ and 0.1% sodium azide. Endogenous biotin was blocked by means of a DakoCytomation blocking kit (DakoCytomation, Denmark). In order to block nonspecific binding, sections were treated with 3% BSA in PBS for 15 min. GM1 ganglioside was detected in liver sections using CTB biotin labelled (Sigma, USA), diluted 1:300 in PBS, plus 3% BSA at 8°C for 16.5 h, followed with streptavidin-peroxidase polymer at room temperature for 1 h. Peroxidase activity was visualized with diaminobenzidine tetrahydrochloride for 20 min in the dark. Sections were mounted in mounting medium Dako S3025 (Dako North America, CA, USA).

Two negative control tests were performed for each group. First, CTB was omitted in immunohistochemical staining. Second, fixed sections were extracted with chloroform : methanol 2:1 at room temperature for 30 minutes followed by standard immunohistochemical staining.

### 2.6. Densitometric Analysis of GM1 Ganglioside in Sinusoidal Membrane and Adjacent Cytoplasm Areas

Six liver specimens were used from each animal. One section from each specimen was used for GM1 ganglioside detection with CTB as described above. From each section, four hepatic lobules with a clearly discernible central vein were selected. In each lobule, one measuring frame in the central lobular zone III and one measuring frame in the corresponding peripheral lobular zone I were selected for analysis. In each frame, 15 areas of sinusoidal surface and 15 areas of adjacent hepatocyte cytoplasm were selected by the stratified random sampling method [24] and marked out. The reaction product was quantified as the mean optical density of the analyzed areas (determined by the densitometric program ACC 6.0, SOFO, Czech Republic) at objective magnification of 40x (NA = 0.7). The ratios of densities measured in the sinusoidal membrane and subsinusoidal intracellular compartment were measured and compared together (sin/int).

### 2.7. Cell Culture Experiments

Human neuroblastoma cell line SH-SY5Y (ATCC, Manassas, VA, USA) was cultured in the Minimum Essential Medium Eagle (MEM) and Ham's F-12 medium (1:1, *v*/*v*) with 15% of fetal bovine serum and human hepatoblastoma cell line HepG2 (ATCC, Manassas, VA, USA) in MEM with 10% of fetal bovine serum in a humidified atmosphere (containing 5% CO_2_ and 37°C). Authentication of used cell lines was confirmed by an independent laboratory using a method based on an accredited short tandem repeat analysis.

Cells were seeded onto 6-well plates (Corning, NY, USA) at a concentration of 50,000 cells per 1 cm^2^ and treated with CDCA and bilirubin for 4 h. SH-SY5Y cells were also treated with PKC activator (phorbol 12-myristate 13-acetate) or PKC inhibitor (Ro 31-0432) for 4 h. After the incubation period, cells were harvested into the lysis buffer and stored at −80°C for further experiments.

### 2.8. Measurement of Intracellular ROS Production

ROS production was determined using a fluorescent probe 5-(and-6)-chloromethyl-2′,7′-dichlorodihydrofluorescein diacetate acetyl ester (CM-H_2_DCFDA, Life Technologies, USA). Briefly, SH-SY5Y cells were grown in 12-well plates to 80% confluence. Cells were then incubated with CDCA and/or antioxidant (bilirubin) for 24 h. After the incubation, the cells were washed twice with PBS and loaded with 10 *μ*M CM-H2DCFDA at 37°C for 30 min in the dark, then washed with PBS to remove excess dye. Fluorescence was measured using 485 nm excitation and 540 nm emission wavelengths in microplate reader (Synergy HTX, BioTek, USA). Cells were then lysed with Cell Lysis Buffer (Cell Signaling Technology, USA), and protein concentration was measured using DC Protein Assay (Bio-Rad, USA) according to the manufacturer's instruction. Data were normalized to protein content and expressed as % of controls.

### 2.9. Lipid Peroxidation

Lipid peroxidation was measured according to the method by Vreman et al. [31]. Twenty microliters of 10% liver or brain sonicates in 0.1 M phosphate buffer, pH = 7.4, were incubated at 37°C with 100 *μ*M ascorbate (80 *μ*L) and 6 *μ*M Fe^2+^ (0.5 *μ*L) in a septum-sealed vial. Butylated hydroxytoluene (10 *μ*M) was added to the blank reaction. The reaction was terminated by adding 2 *μ*L of 60% sulfosalicylic acid. CO produced into the vial headspace was quantified by gas chromatography with a reduction gas analyzer (Peak Laboratories LLC, Mountain View, CA, USA). The amount of CO produced served as an index of lipid peroxidation, was measured as picomoles of CO per hour per milligram of fresh tissue, and was expressed as % of control.

### 2.10. Western Blotting

Cells grown to 60% confluency were lysed using RIPA buffer supplemented with phosphatase and protease inhibitors (Protease Inhibitor Mix G and Phosphatase Inhibitor Mix I, Serva, Heidelberg, Germany). Samples were separated by SDS-PAGE on 12% polyacrylamide gel and then transferred to nitrocellulose membrane (Bio-Rad Laboratories, Hercules, CA, USA). After blocking in Tween-PBS with 5% BSA (Sigma-Aldrich, St. Louis, MO, USA) for at least 1.5 h, membranes were incubated with GM3 synthase and GM2/GD2 synthase antibody (1:2000; Santa Cruz sc-365329 and sc-376505, Dallas, TX, USA), or *β*-actin (1:2000; Cell Signaling Technology, Danvers, MA, USA) overnight at 4°C. After washing, membranes were incubated with anti-mouse m-IgG*κ* BP-HRP (Santa Cruz sc-516102, Dallas, TX, USA) for 1 h. Immunocomplexes on the membranes were visualized with ECL Western Blotting Detection Reagents (Cell Signaling Technology) using an Odyssey infrared imaging system (LI-COR Biosciences, Lincoln, NE, USA).

### 2.11. Quantitative Real-Time PCR

The liver samples were stored frozen at −80°C in RNAlater (Sigma-Aldrich, St. Louis, USA), and total RNA was isolated using a Qiagen RNAeasy Plus kit and QIAshredder (Qiagen, USA). Cell culture samples were stored in lysis buffer at −80°C, and total RNA was isolated using PerfectPure RNA Cell Kit (5Prime, USA). A High-Capacity cDNA Reverse Transcription Kit (Life Technologies, Carlsbad, CA, USA) was used to generate cDNA. Quantitative real-time PCR was performed using TaqMan® Gene Expression Assay Kit (Life Technologies, Carlsbad, CA, USA) for the following genes: *GlcT* (Hs00234293_m1), *GalTI* (Hs00191135_m1), GM3 synthase (*St3GalV*, Mm00488237_m1, and Hs01105379_m1), GD3 synthase (*STSia8*, Mm00456915_m1, and Hs00268157_m1), GM2/GD2 synthase (*B4GalNT1*, Mm00484653_m1, and Hs01110791_g1), and GM1 synthase (*B3GalT4*, Mm00546324_s1, and Hs00534104_s1), all provided by Life Technologies (Carlsbad, CA, USA). The data were normalized to HPRT and expressed as percent of control levels.

### 2.12. Statistical Analysis

Normally distributed data are presented as mean ± SD and analyzed by the Student *t*-test. The Mann–Whitney *U* test or Kruskal-Wallis test were used in skewed data. Differences with *P* < 0.05 were considered significant.

## 3. Results

### 3.1. The Impact of *Hmox1* Knockout on the Liver and Brain Ganglioside Content

To investigate the role of *Hmox1* knockout on the ganglioside pattern, we measured changes in ganglioside composition in the liver as well as the brain, the tissue with the highest glycolipid content *in vivo*. As the ganglioside spectra differ within specific tissues, only major gangliosides and representatives of two main biosynthetic pathways, *a-* and *b-*series, were determined.

**Figure 1:**
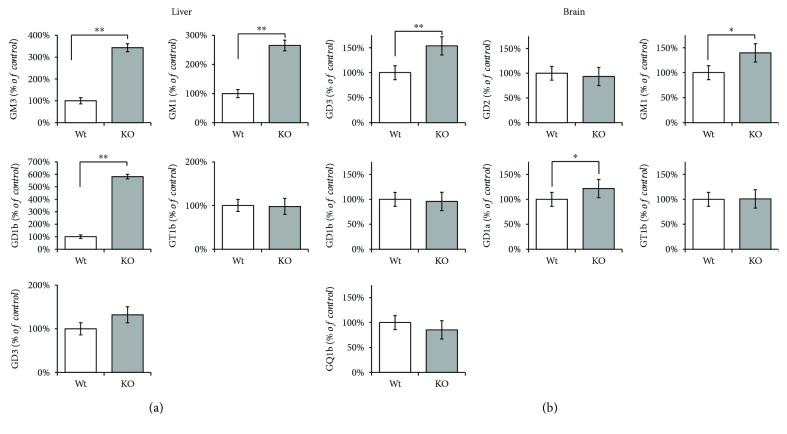
The impact of *Hmox1* knockout on ganglioside composition in mouse (a) liver and (b) brain. Isolated hepatic (a) and brain (b) gangliosides were separated in a solvent system and analyzed by a densitometric method after TLC separation and detection using resorcinol-HCl reagent. Values are expressed as % of control and represent mean SD. Wt: wild-type (*n* = 6); KO: *Hmox1* knockout (*n* = 9). ^∗^*P* < 0.05 and ^∗∗^*P* < 0.01.

In the liver, mice lacking *Hmox1* exhibited marked increases in the concentrations of individual gangliosides. Specifically, *Hmox1* knockout led to a significant increase in GM3 (343 ± 76%, *P* < 0.001) and GM1 (265 ± 62%, *P* < 0.001) representing *a-*series, and GD1b (582 ± 176%, *P* < 0.001) from *b-*series of gangliosides (Figure 1(a)).

**Figure 2:**
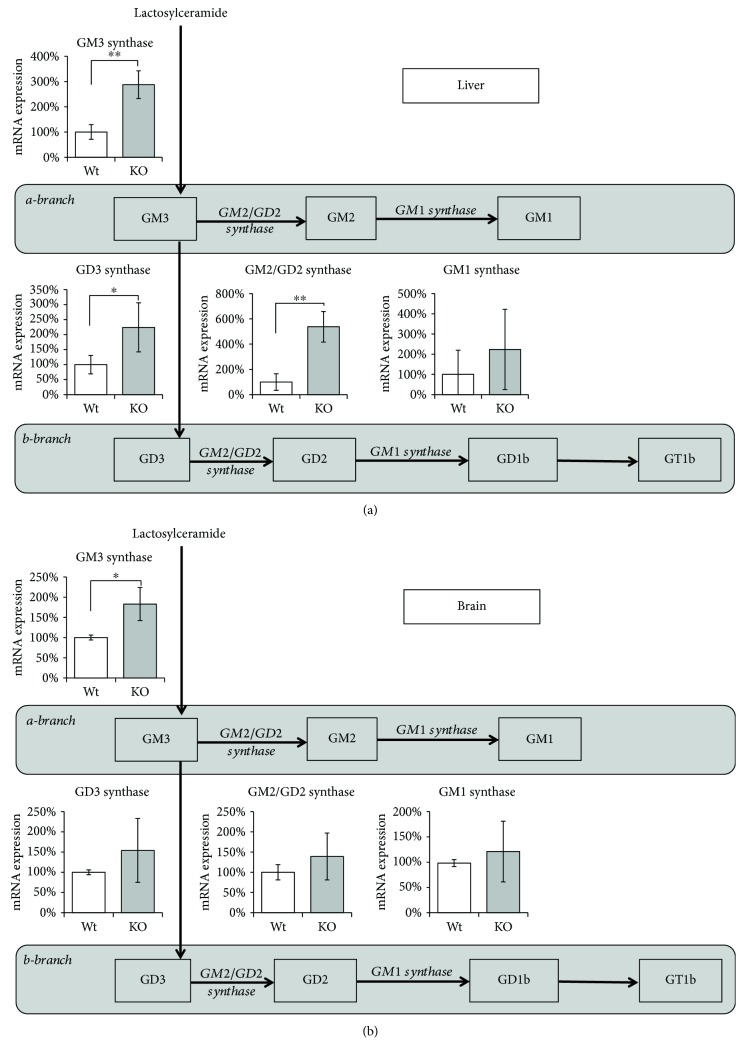
*Hmox1* knockout leads to changes in sialyltransferase expression in the liver and brain. Relative mRNA expression of the key enzymes in ganglioside synthesis was measured in the liver (a) and brain (b) tissues of wild-type (Wt) and *Hmox1* knockout (KO) animals. Values are expressed as % of control. Wt: wild-type (*n* = 6); KO: *Hmox1* knockout (*n* = 9). ^∗^*P* < 0.01; ^∗∗^*P* < 0.001.

In the brain, the most abundant ganglioside was GD1a in both wild-type as well as knockout animals. Together with GM1, GD1a content was significantly higher (GD1a 122% vs. Wt, *P* < 0.05; GM1 140% vs. Wt, *P* < 0.05) in *Hmox1* knockout mice as compared to wild types. The other two major brain gangliosides (GM3, GT1b) stayed unchanged after *Hmox1* knockout. The amount of minor GD3 ganglioside was also significantly increased (154% vs. Wt, *P* < 0.01) (Figure 1(b)). The scheme of de novo biosynthesis of the oligosaccharide moieties of gangliosides is illustrated in Figure 2.

To confirm the level of oxidative stress in *Hmox1* knockouts, we measured the extent of lipid peroxidation in liver and brain tissue homogenates. Importantly, liver lipid peroxidation was increased in *Hmox1* knockout mice as compared to controls (155% ± 51% *Hmox^−/−^*, *n* = 7, vs. 100% ± 46% *Hmox^+/+^*, *n* = 6, *P* = 0.032). No significant increase was observed in a brain tissue (115% ± 46% *Hmox^−/−^*, *n* = 7, vs. 100% ± 45% *Hmox^+/+^*, *n* = 6, *P* = 0.311).

### 3.2. *Hmox1* Knockout Results in Changes in the Expression of Sialyltransferases

Relative mRNA expression of the key sialyltransferases was determined to elucidate the activation rate of *a-* and *b-*series of a ganglioside biosynthetic pathway in mouse liver and brain homogenates. *Hmox1* knockout led to a significant increase in mRNA expression of GM3 synthase (*ST3GalV*) (287 ± 55%, *P* < 0.001; 183 ± 41%, *P* < 0.01) in both liver and brain, and GD3 synthase (*St8SiaI*) (224 ± 89%, *P* < 0.01), the key step in an activation of *b-*biosynthetic branch in the liver. *Hmox1* knockout caused also significant activation of GM2/GD2 synthase (*B4GalNTI*) (538 ± 121%, *P* < 0.001) in the liver. Expression of GM1 synthase (*B3GalTIV*) stayed unchanged in both liver and brain (Figure 2).

### 3.3. *Hmox1* Knockout Leads to a Marked Shift of Gangliosides to the Hepatocyte Membrane

**Figure 3:**
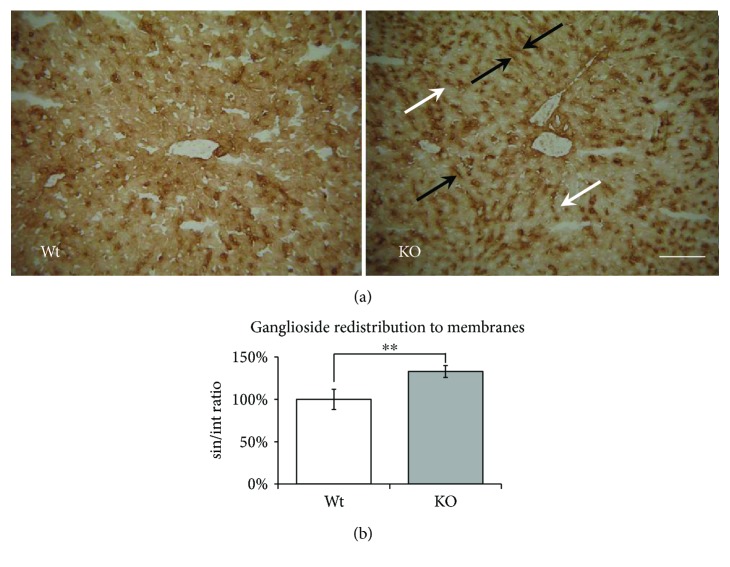
The effect of *Hmox1* knockout on distribution/localization of GM1 ganglioside in the liver. (a) Immunohistochemical detection of GM1 ganglioside. In the liver sections, GM1 ganglioside was detected using the cholera toxin B subunit with streptavidin-peroxidase polymer. Diaminobenzidine tetrahydrochloride (brown colour) was used for visualization. The shift of GM1 ganglioside expression from intracellular localization (white arrows) into sinusoidal membranes (black arrows) was observed in *Hmox1* knockout animals. (b) Quantification of GM1 staining in the liver. Image analysis of the distribution of GM1 ganglioside staining in the subsinusoidal part of the intracellular compartment (int) and sinusoidal membranes (sin) of hepatocytes, expressed as the sin/int ratio relative to control (Wt). The reaction product was quantified as the mean optical density of the analyzed areas at objective magnification of 40x (NA = 0.7). Bar represents 100 *μ*m. Wt: wild-type (*n* = 6); KO: *Hmox1* knockout (*n* = 9). ^∗∗^*P* < 0.01.

To study possible changes in distribution of gangliosides within mouse hepatocytes, histochemical localization of GM1, the representative of gangliosides, was determined in the liver sections. In control liver specimens, GM1 was detected in both sinusoidal and canalicular membranes, as well as in the intracytoplasmic compartment. In *Hmox1* knockout animals, we observed a pronounced shift in GM1 ganglioside expression from intracellular localization into sinusoidal membranes (Figure 3(a)). To quantify this redistribution pattern of GM1, we measured the GM1 expression under high microscopic magnification expressed as sin/int ratio (GM1 staining in the sinusoidal membrane/subsinusoidal intracellular compartment) (133 ± 7%, *P* < 0.01, Figure 3(b)).

### 3.4. Ganglioside Pattern in Neuroblastoma Cells (SH-SY5Y) Is Affected by Oxidative Stress

**Figure 4:**
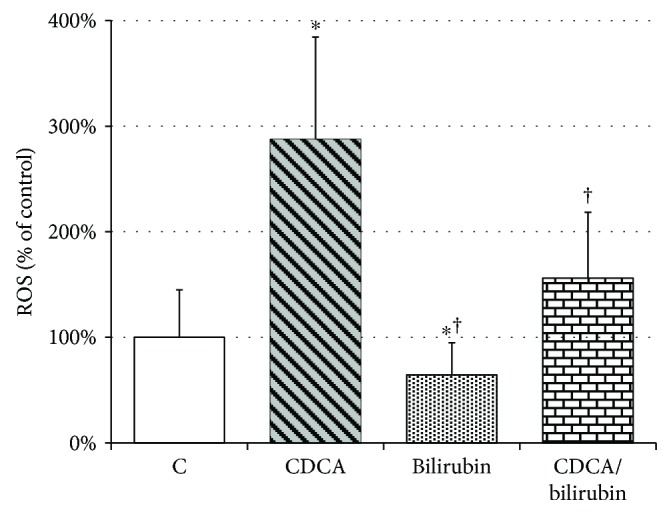
The ameliorating effect of bilirubin on CDCA-induced oxidative stress in SH-SY5Y cells. SH-SY5Y cells were incubated with CDCA (80 *μ*M), bilirubin (1 *μ*M), or both (CDCA/bilirubin) for 24 h. ROS production was measured using fluorescent CM-H2DCFDA probe. Values are expressed as % of controls. C: control; CDCA: chenodeoxycholic acid (80 *μ*M); bilirubin: 1 *μ*M bilirubin; CDCA/bilirubin: CDCA (80 *μ*M) + bilirubin (1 *μ*M). ^∗^*P* < 0.05 vs. C; ^†^*P* < 0.05 vs. CDCA.

To find out whether changes in the ganglioside pattern might be affected by an increased oxidative stress associated with *Hmox1* knockout, we investigated the regulation of glycosphingolipid (GSL) synthesis using SH-SY5Y neuroblastoma cells rich in gangliosides. CDCA, a potent inducer of ROS production accumulating in the liver during cholestasis, was used to increase oxidative stress *in vitro*, while addition of bilirubin, a potent antioxidant and a product of the Hmox pathway, had an opposite effect (Figure 4).

**Figure 5:**
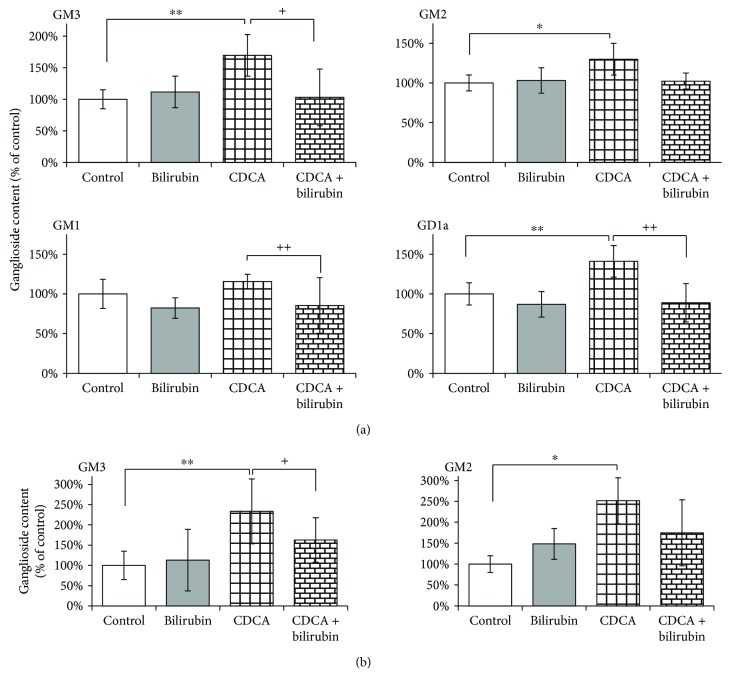
The ameliorating effect of bilirubin on CDCA-induced change in ganglioside content in SH-SY5Y cells (a) and HepG2 cells (b). Relative content of individual gangliosides was evaluated using extraction and TLC densitometry after incubation with CDCA or CDCA/bilirubin for 4 in (a) SH-SY5Y cells and (b) HepG2 cells. Values are expressed as % of controls. Bilirubin (1 *μ*M); CDCA: chenodeoxycholic acid (80 *μ*M); CDCA/bilirubin: CDCA (80 *μ*M) + bilirubin (1 *μ*M). ^∗^*P* < 0.05 vs. C; ^∗∗^*P* < 0.01 vs. C; ^+^*P* < 0.05 vs. CDCA; ^++^*P* < 0.01 vs. CDCA.

Administration of CDCA (80 *μ*M) resulted in a significant increase in the major gangliosides GD1a (141%, *P* < 0.01), GM3 (170%, *P* < 0.01), and GM2 (130%, *P* < 0.05) in SH-SY5Y neuroblastoma cells (Figure 5(a)) and GM3 (233%, *P* < 0.01) and GM2 (251%, *P* < 0.05) in hepatoblastoma HepG2 cells (Figure 5(b)). Interestingly, coadministration of bilirubin (CDCA/bilirubin), a potent antioxidant, resulted in normalization of the ganglioside pattern in both cell lines (Figure 5).

### 3.5. Oxidative Stress-Mediated Changes in Sialyltransferase (*ST3GalV*) Expression Are Regulated through the Protein Kinase C Pathway

**Figure 6:**
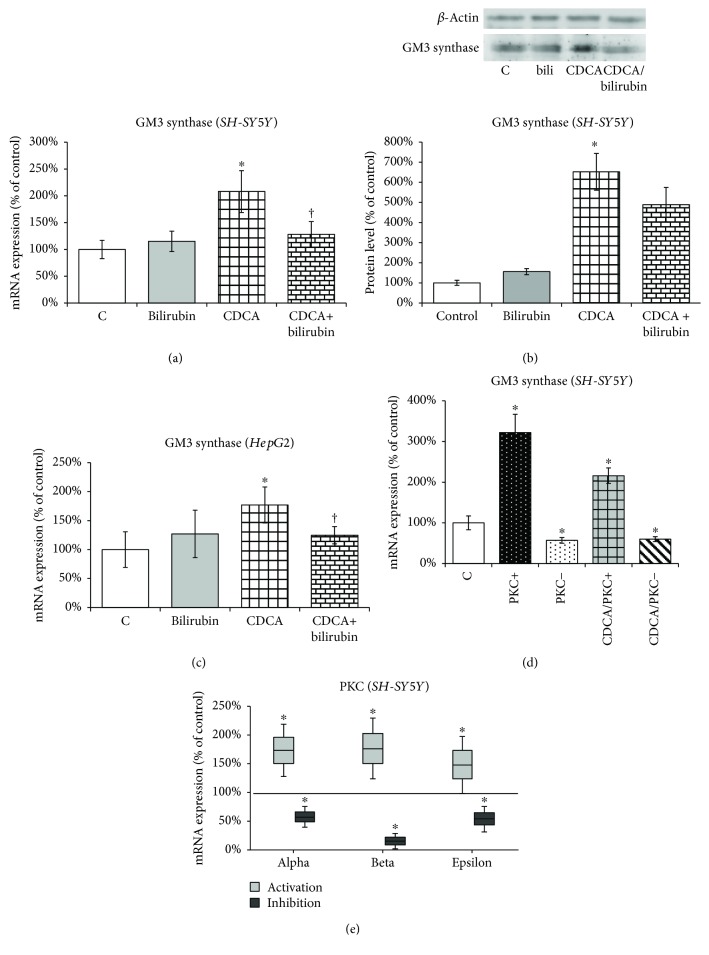
The opposite effects of CDCA and bilirubin on regulation of GM3 synthase expression in SH-SY5Y and HepG2 cells. (a) Relative GM3 synthase (ST3GalV) mRNA expression was determined in control cells (C), after 4 h incubation with chenodeoxycholic acid (CDCA) and/or bilirubin in SH-SY5Y cells. (b) Relative GM3 synthase (ST3GalV) protein expression by Western blot was determined in control cells (C), after 4 h incubation with chenodeoxycholic acid (CDCA) and/or bilirubin in SH-SY5Y cells. (c) Relative GM3 synthase (ST3GalV) mRNA expression was determined in control cells (C), after 4 h incubation with chenodeoxycholic acid (CDCA) and/or bilirubin in HepG2 cells. (d) PKC activity was modulated by incubating SH-SY5Y cells with PKC activator (PKC^+^) or PKC inhibitor (PKC^−^) or their combination with CDCA (CDCA/PKC^+^, CDCA/PKC^−^) for 4 h. (e) PKC activation and/or inhibition was proven by determination of mRNA expression vs. control (100% line) of PKC alpha, PKC beta, and PKC epsilon in SH-SY5Y cells. Values are expressed as % of controls. C: control; PKC^+^: PKC activation by phorbol 12-myristate 13-acetate (5 *μ*M); PKC^−^: PKC inhibition by Ro 31-0432 (5 *μ*M); CDCA: chenodeoxycholic acid (80 *μ*M); CDCA/bilirubin: CDCA (80 *μ*M) + bilirubin (1 *μ*M). ^∗^*P* < 0.05 vs. C; ^†^*P* < 0.05 vs. CDCA.

To elucidate whether oxidative stress induced by CDCA affects the expression of GM3 synthase (*ST3GalV*), a key enzyme in ganglioside metabolism, *in vitro*, SH-SY5Y as well as HepG2 cell lines were incubated with CDCA and/or bilirubin for 4 h. Significant increases in GM3 synthase mRNA expression were observed upon CDCA treatment while cotreatment with bilirubin abolished this effect in both neuroblastoma (Figure 6(a)) and hepatic (Figure 6(c)) cell lines. The results were confirmed by the detection of GM3 synthase protein expression in the SH-SY5Y cell line (Figure 6(b)).

To investigate the possible role of the PKC pathway on oxidative stress-mediated changes in ganglioside expression, we measured the effect of PKC induction/inhibition (PKC^+/−^) on the mRNA expression of *GM3 synthase* (*ST3GalV*), in the SH-SY5Y cell line. PKC activators induced the mRNA expression of *ST3GalV.* On the other hand, PKC inhibitors significantly decreased *ST3GalV* mRNA expression. Importantly, cotreatment of CDCA with PKC inhibitor completely abolished the stimulatory effect of CDCA on *ST3GalV* mRNA (Figure 6(d)). Successful PKC activation and/or inhibition was proven by determination of mRNA expression of PKC alpha, PKC beta, and PKC epsilon (Figure 6(e)).

## 4. Discussion

Gangliosides play a crucial role in signal transduction pathways, regulating many different cell functions such as proliferation, differentiation, adhesion, and cell death [32, 33]. They are responsible for the rigidity of a plasmatic membrane [34] and participate in a protection against oxidative stress [19, 21]. However, the significance of changes in ganglioside metabolism under oxidative stress remains to be elucidated. To address this issue, we have studied the consequences of the antioxidant enzyme Hmox1 deficiency for ganglioside metabolism in mouse tissues. Unlike in the brain, we found significantly increased lipid peroxidation in the liver of *Hmox1* knockout animals which is in accordance with the published data showing increased lipid peroxidation and hepatic injury mostly due to iron accumulation in the liver tissue [6]. Our results indicate that oxidative stress plays an important role in ganglioside synthesis resulting in changes in their spectra and cellular distribution.

Gangliosides are ubiquitously found in tissues and body fluid with the most abundant expression in the nervous system [35]. The expression levels of gangliosides undergo dramatic changes during various physiological and pathological conditions including cell death, proliferation, differentiation, development, and oncogenesis [36–38] as well as neurological diseases [39, 40]. These effects are largely attributed to the changes in expression levels of ganglioside synthases (glycosyltransferases) [41, 42]. In our previous experiments on rats, we observed the shift in liver ganglioside synthesis towards more complex ones in various types of cholestatic liver diseases [25, 30]. These changes were associated with the accumulation of detergent and prooxidative bile acids as well as with the increase in oxidative stress in these animals [21, 22].

In the present study, *Hmox1* knockout resulting in the absence of an important antioxidant enzyme in experimental mice was accompanied by significant increases in the brain GM1, GD1a, and GD3 and liver GM1, GM3, and GD1b gangliosides. The tissue specificity of these changes might be explained by the different ganglioside composition of the liver and brain tissues. While GM3 is the main ganglioside in the liver, GD1a is the most abundant in the adult brain [38, 43], where GD1a, GM1, GD1b, GT1b, and GD3 belong to most important glycosphingolipids [44]. Several reports suggest gangliosides to possess antioxidant properties, but very little is known about the function of gangliosides in the liver and therefore most data relates to the nervous tissue [11]. Among these, GM1 ganglioside has neuroprotective functions. Micelles containing GM1 inhibited iron-catalysed hydroxyl radical formation *in vitro* [18], GM1 decreased ROS formation in rat brain synaptosomes [45], or protected cells against H_2_O_2_-induced oxidative damage [46] while GD1b was able to inhibit lipid peroxidation in human sperm cells [47]. On the other hand, some gangliosides might enhance ROS formation and contribute to the cell death. Sohn et al. [48] found GM3, but not GD3 or GT1b, to mediate oxidative toxicity induced by glutamate in immortalized mouse neuronal HT22 cells. GD3 was described to interact with mitochondria and generate ROS [49, 50], and there is strong evidence for involvement of GD3 in autophagosome formation [51, 52]. GD3 is also considered a key player in apoptosis by Fas, ceramide, and amyloid-*β* [53, 54]. These findings suggest an important role of gangliosides in processes involved in oxidative stress regulation which might explain their compensatory upregulation in the prooxidative environment of *Hmox1* knockout.

The effect of oxidative stress on changes in ganglioside synthesis was further supported by determination of glycosyltransferase mRNA expressions in mouse liver and brain homogenates. The key regulatory enzymes in the synthesis of nearly all gangliosides, *GM3* and *GD3 synthases*, as well as *GalNAcT*, were found to be significantly increased in *Hmox1* knockouts while GM1 synthase expression stayed unchanged. These data correspond with the observed increases in liver gangliosides and are in accordance with our previous observations on liver glycosyltransferase expression in experimental cholestasis in rats [22]. The increase in liver GM1 ganglioside content in *Hmox1* knockouts allows speculating that expression of *GM1 synthase* is redundant in wild-type animals and is capable of maintaining the induction of the *GalNAcT* product. Interestingly, only GM3 synthase has been found to be significantly upregulated in the brain suggesting the tissue-specific regulation of various sialyltransferases. Moreover, different extents of oxidative stress in particular tissues might affect the final sialyltransferase expression.

Furthermore, in our earlier reports, we described not only an increase in ganglioside synthesis but also their shift into the sinusoidal membranes of hepatocytes upon oxidative stress induced by bile acids [21]. This mechanism could protect hepatocytes against detergent and prooxidant effects of bile acids. A very similar effect was observed in the present study. We have used a selective histochemical approach based on the high binding affinity of *Cholera toxin* B subunit to GM1 ganglioside [6], the representative of the complex gangliosides. A significant shift of GM1 gangliosides from intracellular localization to the membrane compartment was found in *Hmox1* knockout which is also associated with prooxidative condition.

To investigate the mechanism of oxidative stress-mediated changes in ganglioside metabolism, we used the *in vitro* model of the SH-SY5Y neuroblastoma cell line rich in glycosphingolipids and, for the comparison, the human hepatoblastoma HepG2 cell line. Interestingly, the HepG2 cell line was found to be very poor in ganglioside content and completely lacking GD3 synthase. Exposure of SH-SY5Y to oxidative stress induced by chenodeoxycholic acid [55] resulted in a significant increase in all major gangliosides of this cell line—GD1a, GM3, and GM2—while addition of a potent antioxidant, bilirubin [56], resulted in normalization of the ganglioside content. Importantly, the same pattern was observed in the HepG2 cell line in GM3 and GM2 gangliosides. These results are in accordance with our earlier observations [10] that bilirubin may counteract a prooxidative effect of BA on hepatocytes in the model of obstructive cholestasis in rats. Furthermore, accumulation of hydrophobic BA in the brain and their possible involvement in hepatic encephalopathy associated with cholestatic liver diseases has been reported [57]. BA can act as cell signalling effectors through binding and activating receptors on both the cell membrane and nucleus. BA signalling encompasses both direct (FXR, TGR5) and indirect (FGF19, GLP-1) pathways. The role of BA in extrahepatic diseases is becoming more important, and increasing amount of reports suggests that BA might play an important role in neurological function and diseases [58, 59].

To elucidate the mechanism of oxidative stress-induced changes of ganglioside metabolism, we focused on regulation of the main enzyme in complex ganglioside synthesis, GM3 synthase (*ST3GalV*).

PKC appeared to be a logical candidate regulating the expression of GM3 synthase. Hydrophobic bile acids are considered potent inducers of PKC while antioxidants inhibit PKC activity [60, 61]. For more than 30 years, it has been known that ganglioside metabolism is in tight connection to PKC activity [62–64], and the action of glycosyltransferases is controlled through posttranslational modification. Glycosyltransferase activities have been demonstrated to be significantly modulated by the action of PKC [65]. Another study suggested the role of PKC as an activator of GM3 synthase (*ST3GalV*) [66]. Our *in vitro* data support this hypothesis. While PKC activators and oxidative stress induced the expression of *ST3GalV*, PKC inhibitors as well as antioxidants completely abolished this effect.

There are some limitations of our study. First, we were primarily interested in the shift of GSL to the cytoplasmic membrane; however, more studies are needed to assess whether subcellular localization and trafficking of gangliosides are affected as well. Second, in histochemical analyses, we used GM1 as a GSL representative but further studies with individual gangliosides are needed to confirm that the shift of GM1 from the intracellular compartment to the cytoplasmic membrane is a general reaction to loss of Hmox1 action. Finally, the PKC pathway is an important but probably not the only pathway regulating GSL metabolism affected by oxidative stress.

## 5. Conclusions

We conclude that oxidative stress is an important factor modulating synthesis and distribution of gangliosides *in vivo* and *in vitro*. Knockout of *Hmox1*, an important antioxidant enzyme, results in tissue-specific increases in main gangliosides together with changes in mRNA expression of key enzymes of ganglioside synthesis. We demonstrate that these changes might be, at least partially, mediated through modulation of the PKC pathway.

## Data Availability

The raw data used to support the findings of this study are available from the corresponding author upon request.
